# Vulvar Langerhans cell histiocytosis: a case report

**DOI:** 10.11604/pamj.2014.18.119.3204

**Published:** 2014-06-06

**Authors:** Nadia Khoummane, Cyriane Guimeya, Dominique Lipombi, François Gielen

**Affiliations:** 1 Department of Gynecology and Obstetrics, Oncology and High Risk Pregnancies- Maternité Souissi, Rabat, Morocco; 2 Université libre de Bruxelles, Department Of Gynecology And Obstetrics, CHR de la Haute Senne, Soignies, Belgium; 3 Department Of Gynecology And Obstetrics, CHR de la Haute Senne, Soignies, Belgium

**Keywords:** Histiocytosis X, Langerhans cell histiocytosis

## Abstract

Langerhans cell histiocytoses (LCH) are a rare group of disorders that comprise a large spectrum of diseases initially known as histiocytosis X. In this case report, we relate a case of LCH affecting the vulva of a 47-year-old female. The patient presented since 3 years with a vulvar lesion characterized by non-healing ulcers and a perineal granuloma on which she underwent surgery. Professionals should keep in mind not to treat straightforwardly lesions of the genital tract as simple sexually transmitted diseases. Chronic, atypical genital lesions seen in women need to be worked up and dealt with accordingly.

## Introduction

Langerhans cell histiocytoses (LCH) are a rare group of disorders that comprise a large spectrum of diseases initially known as histiocytosis X. They present as a clonal neoplastic proliferation of Langerhans type cells. Three main clinical manifestations of Langerhans cell histiocytosis have been identified (Letterer-Siwe disease, Hand-Schuller-Christian disease and Hashimoto-Pritzker disease) but many intermediate and acute forms of the disease have been described. They vary from a local invasion requiring local treatment (60%) to an acute, more disseminated type. Genital LCH as the unique manifestation of this disease is rare. We present a case of a woman with LCH affecting solely the vulva.

## Patient and observation

**Figure 1:**
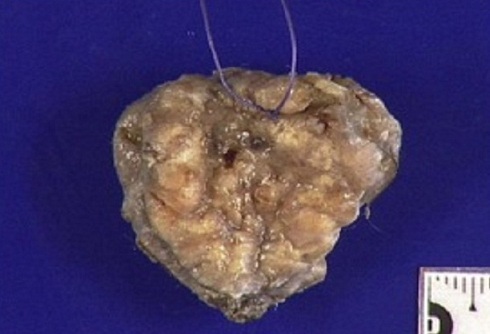
Macroscopic aspect of vulvar langerhans cell histiocytosis

**Figure 2:**
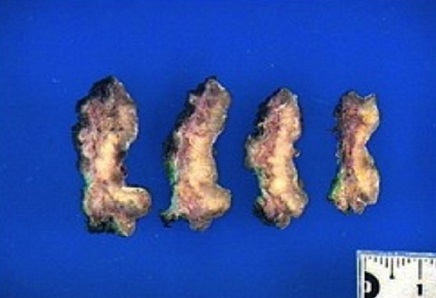
Macroscopic aspect of vulvar langerhans cell histiocytosis

**Figure 3:**
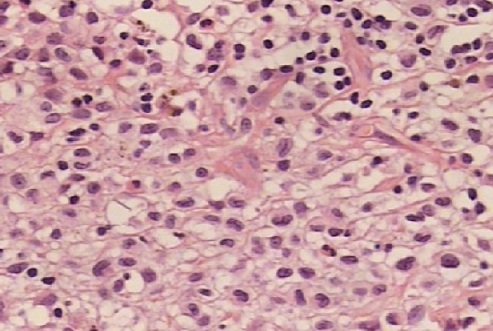
Microscopic aspect of vulvar langerhans cell histiocytosis (lens 40)

**Figure 4:**
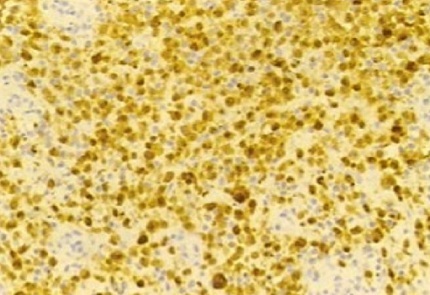
Microscopic aspect of vulvar langerhans cell histiocytosis: immunolabelling CD 1a (lens 20)

**Figure 5:**
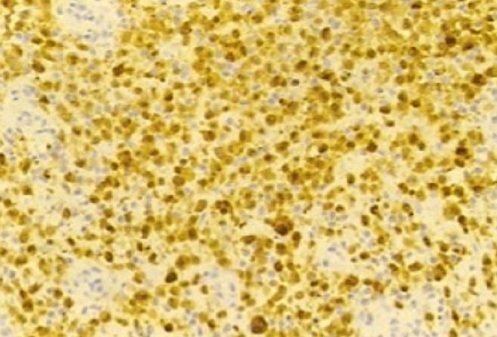
Microscopic aspect of vulvar langerhans cell histiocytosis: immunolabelling S 100 (lens 20)

The patient is a 47 year old, with no significant past medical history, presenting since 2009 with a perineal lesion for which a histopathological examrevealeda perineal granuloma. The patient sought consultation in June 2012 with the same complaint: a vulvar lesion for which she was operated a month later in July 2012. The lesion was excised, sent to the lab and the diagnosis of Langerhans cell histiocytosis surfaced (Figure 1, Figure 2, Figure 3, Figure 4, Figure 5). In this context, in order to determine if the histiocytosis is localized at a gynecological or systemic level, the patient underwent blood tests, a 24h urine collection, a chest and abdominal CT scan and a bone scintigraphy. Blood tests were within normal limits except for a slight eosinophilia (430/mm^3^). Renal function, hemogramme, liver function tests were all normal. We noticed minimal elevation in alkaline phosphatase reaching 260 U/l. Protein electrophoresis was in favor of an inflammation with elevated alpha-2 globulins: erythrocyte sedimentation rate was at 69mm/H with a normal C-reactive protein measuring 0.19mg/dl. Thyroid hormone levels as well as LH, FSH, Prolactin, cortisol, were all within normal limits. Urine osmolarity was normal and chest/abdominal CT scan was unremarkable. Bone scintigraphy did not show any specific lesion. There was no evidence of disease beyond the vulva. After working up the patient, we concluded to the diagnosis of Langerhans cell histiocytosis located in the vulvar area.

## Discussion

Langerhans cells are peripheral dendritic antigen processing cells of bone marrow origin. They are found throughout the body but primarily in the stratum spinosum of the epidermis [1]. They play an important role in localized immune response to antigen.

Langerhans cell histiocytosis (LCH) is a rare disorder characterized by abnormal proliferation of Langerhans cells. The Histiocyte Society classifies LCH as Single System LCH (SS-LCH) or Multisystem LCH (MS-LCH) depending on the extent and localization of disease [2]. For diagnosis to be made, presence of Birbeck granules by electron microscopy, protein S-100 as well as CD1 antibodies in the histiocytic cells of the lesion must be found [3].

The pathogenesis of the disease still remains debatable. Two main theories emerge: neoplasticversus reactive processes. The first is supported by the fact that the proliferation of Langerhans cells appears to be monoclonal in nature due to somatic changes in tumor suppressor genes [4] as well as the positive response to chemotherapeutic drugs. The role of cytokines and interleukins has not yet been understood. TNF-alpha appears to play an important role [5] as well as E-cadherin, whose absence allows dissemination of LCH from a localized lesion in the skin [6]. The second has its roots from the fact that some cases resolve spontaneously [7], the extensive secretion of multiple cytokines by dendritic cells (a phenomenon known as cytokine storm) in the affected tissue, favorable prognosis and relatively good survival rate in patients without organ dysfunction or risk organ involvement [8].

The clinical manifestations of LCH are diverse. They range from non-specific inflammatory responses such as fever, fatigue, to more specific symptoms depending on the organ involved. In our case, genital lesions are mucocutaneous in nature. They are erythematous plaques often ulcerated. Biopsy of the affected area should be performed in order to start adequate management.

Due to the scarcity of reported cases of vulvar Langerhans cell histiocytosis, there are no gold standard or guidelines of treatment. Many regimens have been suggested ranging from local surgery, topical corticosteroids to chemotherapeutic agents [9]. The use of nitrogen mustard has also been reported and produced satisfactory responses [10].

Thalidomide, a hypnosedative drug has been used successfully for treatment of localized LCH such as perianal lesions [11], genital and disseminated skin lesions [12]. An initial dose of 100mg per day is sufficient until the lesions have healed followed by a maintenance dose of 50mg/day [13, 14]. Treatment is not definitive and has been associated with recurrence.

Finally, professionals should keep in mind not to treat straightforwardly lesions of the genital tract as simple sexually transmitted diseases. Chronic, atypical genital lesions seen in women need to be worked up and dealt with accordingly.

## Conclusion

Langerhans cell histiocytosis of the vulva remains a rare disorder with very few described cases in the literature. Two main subtypes are described by the histiocyte society, which are single and multi system LCH [2]. The pathogenesis still remains under investigation. Whether it is neoplastic or reactive is yet to be proved. Exposing the presence of Birbeck granules, protein S-100 and CD1 antibodies makes diagnosis easier. Treatments of Langerhans cell histiocytosis of the vulva are numerous. Up to date, thalidomide is thought to deliver satisfactory results although it does not exclude the risk of recurrence.
